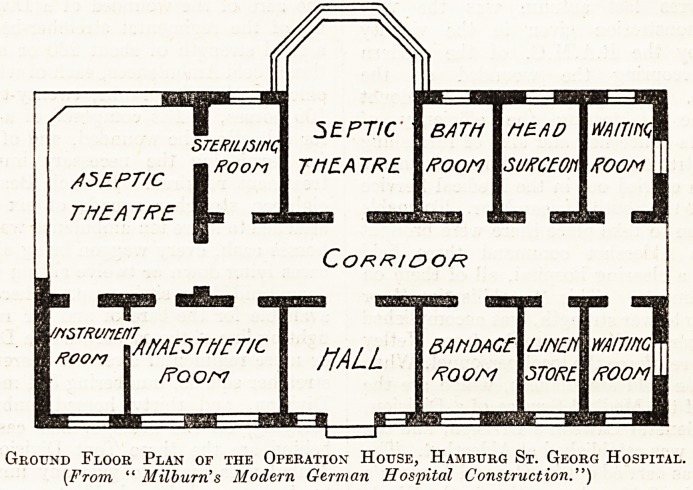# Septic and Aseptic Operating Theatres for Poor-Law Infirmaries

**Published:** 1913-06-21

**Authors:** 


### SEPTIC AND ASEPTIC OPERATING THEATRES FOR POOR-LAW INFIRMARIES.

### The Theory and Practice in England and Germany.

BY AN INFIRMARY SUPERINTENDENT.

IN a recent number of THE HOSPITAL attention was drawn to the special requirements of the Poor-Law infirmary as regards the provision of an operating room for septic cases. The matter, however, is one of wider extent, and a brief account of the arrangements in the leading hospitals to-day may be of some interest.

As regards theoretical considerations there is a general agreement amongst the chief writers of recent years on aseptic surgery and hospital construction. Practically all of them advocate the provision of separate rooms for septic and aseptic operations. Lockwood [1] says: "As far as possible all septic cases should be operated upon in a theatre kept for the purpose." Burghard [2] goes further. He states: "For septic cases separate wards and theatres should be set apart." Depage [3] quotes Schönborn, Neuber, and Terrier as advocating the necessity for separate theatres for septic and aseptic cases and adds: "With all our colleagues in the hospitals of Brussels we think that the operating suite must include three rooms—(1) for aseptic cases; (2) for septic cases; (3) for infectious cases." He states that Neuber considers that in the absence of such separation all antiseptic methods or sterilisation may be considered illusory. Professor Tillmanns [4] writes: "In larger hospitals it is desirable there should be two operation rooms at the disposal of the surgeon, the one for aseptic, the other for septic operations." Ruppell says: "If at all possible special rooms should be provided for aseptic and septic operations. These should be completely separate the one from the other." [5]

With this universal insistence upon the advantages of having a separate theatre for septic cases, it is remarkable how few British hospitals have made such provision. Many hospitals have been to great expense in providing apparatus to filter from the air comparatively innocuous organisms before admission to the theatre, and special floors and walls which can be easily cleaned; but the simplest means of all for preserving the purity of the air and surfaces, the exclusion of cases likely to contaminate them grossly, has been very generally neglected.

GROUND FLOOR PLAN OF THE OPERATION HOUSE, HAMBURG ST. GEORG HOSPITAL.
(*From "Milburn's Modern German Hospital Construction."*)

### THE PRACTICE OF HOSPITALS WITH MEDICAL SCHOOLS.

The need for taking precautions in this respect is, however, becoming more widely realised, and several hospitals have recently built septic theatres or have the matter under consideration. Inquiries made at all the hospitals with medical schools in Great Britain show the following results: Of the twelve hospitals in London, four have separate theatres for septic cases; of the twelve provincial hospitals, two; of the five hospitals in Scotland, three; and of the two in Ireland, neither, but one of them has the matter under consideration. In nearly all the hospitals unprovided with special accommodation, however, it is the rule to take the septic cases at the end of the aseptic operations, and then, if possible, to leave the theatre unused until the following day or even longer, any emergency operation being performed in one of the other theatres. In two of the hospitals all septic opera-

[1] *Aseptic Surgery*, 1909 Edition, p. 173.

[2] *System of Operative Surgery*, 1909 Edition, p. 8.

[3] Depage, *La construction des hôpitaux*, pp. 263-4.

[4] *Lehrbuch der allgemeinen Chirurgie*, p. 22.

[5] Lenharz and Ruppell, *Der moderne Krankenhausbau.*

tions are, if possible, performed in the wards; in another they are performed in a small theatre used for operations on the nose, ear, and throat; and in a fourth in the out-patient operating room. It is obvious that all these methods are makeshifts and will not stand criticism. In Germany the conditions are very different. There in all the modern hospitals special septic theatres are provided as a matter of course. The plan above, copied in a simplified form from "Milburn's Modern German Hospital Construction," shows the kind of provision which is usually made.

The septic theatre is cut off from the part of the block devoted to aseptic operations by means of a glass screen across the corridor. Incidentally this plan shows the provision which is nearly always made in the German operation unit for the comfort of the surgeon in the presence of a bath room and a dressing room for his personal use. The two theatres are always kept strictly separated from one another, and great care is taken in the selection of the cases sent to each. The Düsseldorf General Hospital and the Virchow Hospital, Berlin, have similar operation units.

## Figures and Tables

**Figure f1:**